# Central Nervous System Demyelination Associated With Immune Checkpoint Inhibitors: Review of the Literature

**DOI:** 10.3389/fneur.2020.538695

**Published:** 2020-12-11

**Authors:** Marcos C. B. Oliveira, Marcelo H. de Brito, Mateus M. Simabukuro

**Affiliations:** ^1^Neurology Unit, Instituto do Câncer do Estado de São Paulo (ICESP), Faculdade de Medicina da Universidade de São Paulo (FMUSP), São Paulo, Brazil; ^2^Department of Neurology, Hospital das Clínicas, Faculdade de Medicina da Universidade de São Paulo (FMUSP), São Paulo, Brazil

**Keywords:** demyelination, cancer immunotherapy, anti-PD-L1, anti-CTLA-4, anti-PD-1, immune-related neurological adverse events, immune checkpoint inhibitors (ICI)

## Abstract

Immune checkpoint inhibitors (ICI) are a novel class of antineoplastic treatment that enhances immunity against tumors. They are associated with immune adverse events, and several neurological syndromes have been described, including multiple sclerosis and atypical demyelination. We performed a systematic literature review of case reports with neurological immune adverse events that presented with central nervous system demyelination, up to December 2019. We found 23 cases: seven with myelitis, four isolated optic neuritis, one neuromyelitis optica spectrum disorder, five multiple sclerosis, and six with atypical demyelination. Ipilimumab was the most frequently used ICI (11/23). The median time to develop symptoms from the onset of ICI was 6.5 weeks [range 1.0–43.0], and from last ICI dose was 14 days [range 0–161]. Anatomopathological examination was performed in four cases, with the finding of a T-cell mediated immune response. Outcomes were generally favorable after immunosuppression: 18 patients had improvement or a full recovery, three patients did not respond to treatment, three patients died, and in one, treatment was not reported. We describe the patients' clinical presentation, treatment administered, and outcomes. We further speculate on possible pathophysiological mechanisms and discuss potential treatments that may be worth investigating.

## Introduction

Immune checkpoint inhibitor (ICI) is a novel class of antineoplastic drugs that enhance antitumor immune responses through the upregulation of T cell activity. Their mechanism consists of blocking receptors that normally inhibit the T cell response, the so-called inhibitory immune checkpoints. The main targets of these medications are cytotoxic T lymphocyte antigen 4 (CTLA-4) receptor, programmed cell death 1 (PD-1) receptor, and programmed cell death 1 ligand (PD-L1) (1), which are molecules that ultimately break the T cell immune-mediated response. CTLA-4 is expressed on activated CD4^+^ T helper cells, regulatory T cells, and CD8^+^ cytotoxic T lymphocytes; they bind to its ligands, CD80 and CD86, expressed on professional antigen-presenting cells (APCs) (2). PD-1 is predominantly expressed on T cells—but also in B cells, natural killer cells, and macrophages—and bind to PD-L1, expressed by professional and non-professional APCs (including some tumor cells) (3). Specific monoclonal antibodies that block the inhibitory action of these checkpoint molecules lead to persistent and generalized activation of the humoral and cellular adaptative immune system, enhancing antitumor immunity (4).

ICIs have shown clinically effective antitumor response and improved survival for melanoma, non-small cell lung cancer (NSCLC), renal cell carcinoma, as for an increasing number of other indications. Six of them are currently available in clinical practice: pembrolizumab and nivolumab (anti-PD-1); atezolizumab, avelumab, durvalumab (anti-PD-L1); and ipilimumab (anti-CTLA-4) (1). However, because of their effect in activating the immune system, they are associated with immune-related adverse events (irAE). The most common irAEs are reactions involving the gastrointestinal tract, endocrine glands, skin, and liver (5). Most of them are mild and can be treated with symptomatic medications, but some require interruption or discontinuation of the ICI and the use of IV steroids or other immunosuppressive drugs (i.e., infliximab for colitis) (6).

Although less common, neurologic irAEs (nirAE) may be severe and require prompt recognition and treatment (7). The incidence of high-grade nirAE in clinical trials was <1% in a review study and was slightly more common for anti-CTLA-4 (0.7%) and combined anti-CTLA-4 plus anti-PD-1 (0.7%) than for anti-PD-1 treatment (0.4%) (7). Headache, encephalopathy, meningitis, Guillain Barré-like syndrome, peripheral neuropathy, and myasthenic syndrome were the most common events reported. Several cases of paraneoplastic neurologic syndromes, with or without demonstration of autoantibodies (4), have also been reported, including anti-NMDA (8, 9), anti-Ma2 (10, 11), anti-SOX1 (8), anti-Ri (9), anti-CASPR2 (12), anti-GAD65 (13) encephalitis, anti-Hu sensory neuronopathy, encephalomyelitis and/or limbic encephalitis (9, 14, 15), and myasthenia gravis (11, 16–20).

Worsening or development of multiple sclerosis (MS) associated with ICIs has previously been reviewed in a study using the United States Food and Drug Administration Adverse Event Reporting System (FAERS) data (21). They found 13 MS cases amongst 42,529 reported adverse events plus one from their institution, with five of them having more detailed clinical data published in case reports (21–25). History of MS was confirmed in 8 (57%) cases, the median time to the beginning of symptoms was 29 days, two patients died because of their relapse, and there was no difference in outcomes between CTLA-4 and PD-1/PD-L1 inhibitors. We systematically assessed published cases of demyelinating syndromes in the central nervous system (CNS) associated with ICIs, including cases not classified as MS. This article includes 14 additional new case reports published since 2018, not discussed in previous reviews (7, 21, 26), and focuses on the clinical manifestations, outcomes, and possible mechanisms involved in ICI-associated demyelination.

## Materials and Methods

We performed a systematic literature search on PubMed up to December 2019 mentioning treatment with immune checkpoint inhibitors and demyelinating conditions, using the terms: “demyelination or multiple sclerosis or white matter or optic neuritis or encephalomyelitis or myelitis” combined with “anti-CTLA4 or anti-CTLA-4 or anti-PD1 or anti-PD-1 or ipilimumab or tremelimumab or nivolumab or pembrolizumab or lambrolizumab or pidilizumab or durvalumab or avelumab or atezolizumab.” Additional articles were identified from other sources (i.e., articles cited in reviews). Two investigators (MCBO and MHB) performed the search and collected the data independently for internal validity. We selected published case reports of CNS demyelinating conditions (including optic neuritis) that were temporally related to ICI use, regardless of the time. We relied mainly on the opinion of the reports' authors that the demyelination was ICI-associated. We defined the presence of demyelination based on the description of imaging studies, the authors' interpretation of these studies in the reports, and/or findings on anatomopathological studies. Cases of CNS encephalitis without evidence of demyelination, with imaging suggestive of vasculitis, and without detailed clinical data were excluded.

Primary cancer, treatment regimen, patients characteristics, clinical manifestations of neurological disorder, MRI, CSF, and other tests, other immune-related adverse events (irAE), time to development of neurological symptoms from ICI start and last ICI dose, oncologic response after ICI treatment, antibodies tested, treatment of nirAE, response to treatment and cessation of ICI were noted. Patients were classified into a “clinical syndrome” based on reported previous diagnosis, clinical and imaging features, and current diagnostic criteria. Cases that did not fulfill criteria for multiple sclerosis (27), optic neuritis (28), neuromyelitis optica spectrum disorder (NMOSD) (29), and demyelinating myelitis (30) were classified as “atypical demyelination.” We included in this last group cases that would fit into an encephalomyelitis clinical picture (31, 32). A descriptive statistical analysis was performed for demographic and clinical data. This review is reported according to the PRISMA (Preferred Reporting Items for Systematic Reviews and Meta-Analyses) guidelines (33).

## Results

We found 96 articles through the PubMed search and an additional 54 articles through other sources. We excluded 92 articles after screening the title and abstract. From the 58 articles assessed for eligibility, 25 articles were excluded because they did not comprise a case report with CNS demyelination (Supplementary Figure 1). Articles included in this review contained 23 case reports of patients who developed CNS demyelination after ICI administration. Of these cases, patients were classified as having the following syndromes: 7 with myelitis (19, 34–39), four isolated optic neuritis (40–43), one NMOSD (44), three had a relapse from a previously diagnosed MS (21, 23, 24), and two evolved from a radiologically isolated syndrome (RIS) to MS (22, 45). Six patients had atypical demyelination (14, 25, 46–49). Class of ICI used was anti-PD-L1 in four patients, anti-CTLA-4 in eight, anti-PD-1 in eight, and a combination of anti-PD-1 and anti-CTLA-4 in three patients (in one of them used concomitantly); ipilimumab was the most frequently used ICI (11/23). Patients had a median age of 59 years old [range: 9–75]; 8 of 23 patients were female; median time to development of symptoms from the onset of ICI was 6.5 weeks [range 1.0–43.0], and from last ICI dose was 14 days [range 0–161]; seven of them had other non-neurological irAE reported. Seventeen cases had oncologic outcomes after ICI treatment reported: six partially remitted, five completely remitted, and six had a progression of the oncologic disease (Table 1).

**Table 1:** Clinical syndromes of demyelination and demographic and oncologic data.

	**Clinical syndrome**	**Drug (number of cycles)**	**Age**	**Gender**	**Primary cancer**	**TTO**	**ILD**	**Other irAE**	**Cancer outcome after ICI**	**Reference**
**MULTIPLE SCLEROSIS**										
1	MS relapse	Atezolizumab (1)	49	F	Colon adenocarcinoma	2w	2w	–	PD	(21)
2	MS Relapse^e^	Ipilimumab (NR)	56	M	Melanoma	4w	NR	–	CR	(23)
3	MS Relapse	Nivolumab (1)	42	F	Lung adenocarcinoma	1w	1w	–	NR	(24)
4	Evolution from RIS to MS	Ipilimumab (4)	29	M	Melanoma	16w	7w	Hypophysitis	PR	(22)
5	Evolution from RIS to MS	Pembrolizumab (14)	67	F	Lung adenocarcinoma	43w	NR	–	PR	(45)
**MYELITIS**										
6	Myelitis	Atezolizumab (3)	63	F	Small cell lung cancer	NR	NR	–	NR	(38)
7	Myelitis^c^	Durvalumab (3)	69	M	Lung adenocarcinoma	4w	days	–	NR	(34)
8	Myelitis^c^	Ipilimumab (2)	58	M	Melanoma	26w	23w	–	CR PD	(37)
9	Myelitis	Ipilimumab (3)	62	M	Melanoma	7w	4d	Uveitis, dermatitis, colitis, genitourinary symptoms, acute renal failure	PD	(19)
10	Myelitis^c^	Ipilimumab + Nivolumab –> Pembrolizumab (NR)	68	M	Melanoma	2w^b^	2w	–	PD	(35)
11	Myelitis^c^	Pembrolizumab (8)	68	M	Lung adenocarcinoma	24w	NR	–	PR	(36)
12	Myelitis	Ipilimumab (3)	39	F	Melanoma	7w	days	Hypophysitis	CR	(39)
**NMOSD**										
13	NMOSD	Nivolumab (1)	75	M	Lung squamous cell carcinoma	8w	8w	–	PD	(44)
**OPTIC NEURITIS**										
14	Optic neuritis	Atezolizumab (1)	53	M	Lung adenocarcinoma	3w	3w	–	PR	(41)
15	Optic neuritis	Ipilimumab (3)	53	M	Melanoma	21w	15w	Skin rashes, colitis, Hypophysitis	PR	(43)
16	Optic neuritis	Ipilimumab (4)	70	M	Melanoma	12w	NR	Anterior uveitis	NR	(40)
17	Optic neuritis	Nivolumab (2)	9	M	Glioblastoma multiforme	2w	2d	–	NR	(42)
**ATYPICAL DEMYELINATION**										
18	Brain demyelination^d^	Ipilimumab (4)	76	F	Melanoma	17w	6w	–	PR	(25)
19	Brain demyelination^d^	Ipilimumab (1) –> Nivolumab (11)	44	M	Melanoma	38w^a^	NR	Colitis (after 1 infusion of ipilimumab, treated with infliximab), skin rash (with nivolumab)	CR	(48)
20	Demyelinating encephalitis	Ipilimumab (4) –> Nivolumab (4)	60	M	Melanoma	6w^a^	2d	–	PD	(47)
21	Brain and spine demyelination	Pembrolizumab (3)	58	F	Melanoma	6w	NR	Thyroiditis	CR	(46)
22	Brain demyelination	Nivolumab (1)	59	F	Laryngeal squamous cell carcinoma	2w	2w	–	NR	(49)
23	Anti-Hu encephalomyelitis	Nivolumab (3)	61	M	Lung pleomorphic carcinoma	5w	5d	–	PD	(14)

*TTO, Time to onset of neurologic immune-related adverse event (nirAE) during immune checkpoint inhibitor (ICI) treatment; ILD, Interval from last ICI dose to nirAE; irAE, immune-related adverse event; NR, not reported; w, weeks; d, days; MS, multiple sclerosis; RIS, radiological isolated syndrome; NMOSD, neuromyelitis optica spectrum disorder; +, combination of ICIs; –>, followed by other ICI; PD, progression of oncologic disease; CR, complete remission; PR, partial remission*.

a From the onset of nivolumab;

b From the onset of pembrolizumab;

c Exposure of spinal cord to radiotherapy before ICI;

d Treatment for brain metastases with stereotactic radiosurgery before and/or during ICI;

e *Previously with stable relapsing-remitting multiple sclerosis, glatiramer, and methotrexate were stopped when ipilimumab started*.

All patients but one were investigated with MRI, and anatomopathological examination was performed in four patients (two biopsies and two autopsies). CSF exam was reported in 18 patients, with elevated protein being the most common finding (14/18 cases; median protein of 93.5 mg/dL; range: 50–380), followed by pleocytosis (10/18 cases; median white blood count = 22 cells/mm^3^; range: 14–1,195); oligoclonal bands (OCB) were reported positive in seven patients. Anti-aquaporin4 (anti-AQP4) antibodies were tested in four patients and were positive in one; a paraneoplastic panel was assessed in nine patients, with a positive result in two of them (anti-Hu and anti-CRMP5); one patient was negative for anti-myelin oligodendrocyte glycoprotein (anti-MOG) antibodies. ICI treatment was at least temporarily discontinued in 20 of 23 patients because of the nirAE; in two patients, ICI treatment was maintained because of good oncologic response and benefits overweighting the risks (23, 45); in two cases, ICI treatment was reinstituted after the resolution of nirAE without the development of new irAE (22, 36). Treatment of demyelination included systemic steroids (21/23), plasma exchange (PLEX) (5/23), intravenous immunoglobulin (IVIg) (4/23), infliximab (2/23), interferon (2/23), cyclophosphamide (2/23), glatiramer (1/23), and mycophenolate mofetil (1/23); two patients received no systemic treatment other than discontinuation of ICI, and in one patient treatment was not reported. Outcomes of demyelination were reported in all cases but one: 14 patients had improvement, four patients had a full recovery, three did not respond to treatment, and three died (Table 2).

**Table 2:** Paraclinical information and treatment outcomes of demyelination cases.

	**MRI**	**CSF**	**Other tests**	**Antibodies**	**Treatment**	**Interruption of ICI**	**Outcome**	**Reference**
**MULTIPLE SCLEROSIS**								
1	Hyperintensities within the subcortical, deep, and periventricular white matter	Elevated protein	NR	NR	Steroids, glatiramer	Yes	No response, died	(21)
2	New enhancing lesion consistent with active demyelination	NR	NR	NA	Steroids, glatiramer restarted	No	Improvement	(23)
3	New hyperintense pons lesion with incomplete ring enhancement	NR	NR	NA	Steroids	NR	Full recovery	(24)
4	Multiple white matter hyperintensities with enhancement	WBC = 15, Prot 50, Positive OCB	Biopsy: active MS, T-cell type (pattern 1)	NA	Steroids, interferon	Yes^a^	Improvement	(22)
5	Multiple white matter hyperintensities, nodular spinal cord enhancing lesion	Positive OCB	VEP: increase of latency in both eyes; SSEP: increased cortical latency for stimulation of limbs	NA	Steroids, interferon	No	Improvement	(45)
**MYELITIS**								
6	Extensive thoracic spinal cord lateral tracts hyperintensity with contrast enhancement	WBC = 46, Prot = 105	NR	Anti-CRMP5 positive: 1:3840 (serum) and 1:1024 (CSF)	Steroids, cyclophosphamide	Yes	Improvement	(38)
7	T5–T8 Spinal cord hyperintensity	Normal	NR	NA	Steroids	Yes	Improvement	(34)
8	T7–L1 Spinal cord hyperintensities	WBC = 16, prot = 57	NR	Negative anti-AQP4 and paraneoplastic panel	Steroids, IVIg	Yes	No response	(37)
9	T9–T10 Spinal cord hyperintensity	WBC = 28, Prot = 50	NR	NA	Steroids	Yes	Improvement	(19)
10	T5–T10 Spinal cord hyperintensity with patchy enhancement	Prot = 99, positive (matched) OCB, MBP = 31.6	NR	Negative anti-AQP4 and paraneoplastic panel	Steroids, plasma exchange, cyclophosphamide, infliximab	Yes	Improvement	(35)
11	T12–L1 Spinal cord edema, patchy gadolinium enhancement	Prot = 84	NR	Negative antineural antibodies	Steroids	Yes^c^	Full recovery	(36)
12	Leptomeningeal and cranial nerve enhancement; extensive cervical spinal cord hyperintensities with enhancement	Lymphocytic pleocytosis, Prot = 120	NR	Negative paraneoplastic panel	Steroids, IVIg, Infliximab	Yes	Almost full recovery	(39)
**NMOSD**								
13	Spinal cord hyperintensities	WBC=1195 (53% neutrophils), Prot = 380, Gluc = 40	NR	Anti-AQP4 positive, anti-MOG negative, negative paraneoplastic antibodies panel	Steroids, plasma exchange	Yes	Improvement	(44)
**OPTIC NEURITIS**								
14	Unremarkable	NR	NR	NA	Steroids	Yes	Improvement	(41)
15	Optic nerve enhancement	WBC = 62, Prot = 105	NR	NA	Steroids, mycophenolate mofetil, plasma exchange	Yes	Improvement	(43)
16	NR	Normal	NR	NA	Topical corticosteroids	Yes	Spontaneous improvement	(40)
17	Bilateral thickening of the optic nerves	NR	NR	NA	Steroids	Yes	Full recovery	(42)
**ATYPICAL DEMYELINATION**								
18	Optic nerve and white matter hyperintensities	NR	Biopsy: acute/subacute inflammatory demyelination, myelin reactive T cells	NA	Steroids, Cyclophosphamide	Yes	No response, died	(25)
19	Multiple hyperintense lesions with incomplete ring enhancement, Dawson's fingers	WBC=14, Prot=59, Negative OCB	NR	Negative paraneoplastic panel	None other than discontinuation of nivolumab	Yes	Full recovery	(48)
20	White matter lesions consistent with tumefactive demyelination	WBC = 0, Prot = 88, positive OCB, MBP = 11.0	Autopsy: widespread white matter demyelination	NA	Steroids, IVIg	Yes	Transitory response, died	(47)
21	Multiple periventricular white matter hyperintensities, multiple spinal cord hyperintensities, one of them with gadolinium enhancement	Elevated protein, positive CSF OCB	NCS: normal; SSEP: absence of responses in the limbs	Negative anti-AQP4 and paraneoplastic antibodies panel	Steroids, Plasma exchange	Yes	Almost full recovery	(46)
22	Multiple white matter hyperintensities	WBC = 74, elevated protein, positive OCB	EEG: diffuse generalized slowing	NA	Steroids, IVIg	Yes	Improvement	(49)
23	Temporal, thalamus, cerebral aqueduct, spinal cord hyperintensities	WBC = 16, prot = 162, positive OCB	Autopsy: CD8-positive T cells and macrophages infiltrate, microglia activation	Serum: Anti-Hu antibodies^b^	Steroids, plasma exchange	Yes	No response	(14)

*MRI, magnetic resonance imaging; CSF, cerebrospinal fluid; ICI, immune checkpoint inhibitors; WBC, white blood count (in cells/mm^3^); prot, protein (in mg/dL); gluc, glucose (in mg/dL); MBP, myelin basic protein (in ng/mL); OCB, oligoclonal bands; EEG, electroencephalogram; NCS, nerve conduction study; SSEP, somatosensory evoked potentials; VEP, visual evoked potentials; Anti-AQP4, aquaporin4 antibodies; Anti-MOG, myelin oligodendrocyte glycoprotein antibodies; NR, not reported; NA, not assessed*.

a A new cycle of ipilimumab was administered 1 year after the first cycle;

b present before treatment,

c *Interrupted initially, but after clinical improvement of neurological symptoms and pulmonary progression, within 14 weeks, pembrolizumab was reintroduced*.

### Multiple Sclerosis

We found two distinct patterns of MS patients on reports of ICI-associated demyelination: (1) three patients already diagnosed with the disease who had a relapse during ICI use, and (2) two patients with RIS [i.e., with demyelinating lesions highly suggestive of MS but without clinical symptoms of the disease (50)] who developed symptoms after the use of ICI and then fulfilled criteria for the diagnosis of MS [according to the 2017 revised McDonald criteria (27)]. Except for patient 1 (21), who had encephalopathic symptoms in her relapse, clinical, and imaging characteristics were typical of multiple sclerosis events in both groups, with no pattern suggesting a different mechanism due to ICI use.

Patient 1 (21) was known to have MS when she was started on atezolizumab; glatiramer was maintained during treatment. She developed atypical symptoms for MS, such as fever and confusion, and MRI showed nonspecific T2 hyperintense lesions within the subcortical, deep, and periventricular white matter. She received the presumptive diagnosis of MS relapse based on her history, but she had no improvement despite high dose steroids treatment. Patient 2 (23) had a previous diagnosis of MS, and MS treatment was withheld before ipilimumab started; she subsequently relapsed after treatment and had a good response with steroids and reintroduction of glatiramer. Patient 3 (24) had a lung adenocarcinoma metastatic to the brain and received whole-brain radiotherapy. She had untreated white matter hyperintensities on MRI suggestive of MS and also relapsed after nivolumab; she had a full recovery with high dose steroids.

Patients 4 (22) and 5 (45) had MRI demyelinating white matter lesions without symptoms, fulfilling RIS criteria. Patient 5 developed an enhancing spinal cord demyelinating lesion several months after treatment with pembrolizumab; she was improved after high dose steroids and had no new relapses after interferon-beta treatment, even though ICI was not discontinued. Patient 4 developed new symptomatic periventricular enhancing demyelinating lesions after the second cycle of ipilimumab, followed by optic neuritis. He improved after high dose steroids, and his lesions remained stable with interferon beta-1a treatment. A biopsy was performed in one of the white matter lesions and was compatible with pattern 1 (T cell type) MS. A next-generation analysis of the T cell receptor repertoire was compared between the primary melanoma histology and CSF collected 5 months and 1 year after ipilimumab infusions. They found distinct clonal expansions of CD4^+^ and CD8^+^ T cells in the melanoma and CSF, with considerable overlap between the T cell receptor repertoire in the tumor and the first, but not second CSF sample. They concluded that antitumor response and the inadvertent anti-CNS autoimmune response were directed against different antigens, and therefore, composed of distinct T cell receptor clonotypes. They further hypothesized that activated, tumor-specific T cells transiently entered the CNS compartment, possibly acting as autoaggressive effectors (22).

### Myelitis

We found seven case reports of myelitis associated with ICI. In four of them (34–37), patients were exposed to radiotherapy on the cervical spine for bone metastases before (cases 7, 8, and 11) or during (case 10) ICI treatment. In all cases, delayed radiation myelopathy (DRM) was considered, but some features implicated an immune etiology, at least superimposed. In all four cases, the total dose of radiation was <30 Gy, which is not usually associated with myelopathy. Time was also more compatible with a complication of ICI treatment than with DRM, which is usually a late (more than 6 months) complication of radiotherapy. In two cases (8 and 10), the myelitis extension was much wider than the area exposed to radiation.

Moreover, in three cases (8, 10, and 11), CSF had findings suggesting an inflammatory process: pleocytosis in case 8, positive oligoclonal bands (OCB) in case 10, and high protein in all. Patient 8 progressed despite steroid and IVIg treatment. Patient 10 had a progression in the extension of the myelitis despite treatment with steroids, PLEX, cyclophosphamide, and improved after infliximab administration. Interestingly, in case 11, there was an improvement of myelitis with oral steroids, after which pembrolizumab was rechallenged without new relapses.

Patient 12 (39) presented with lymphocytic meningitis without malignant cells and nodular leptomeningeal enhancement days after the third infusion of ipilimumab and was treated with steroids. A few months later, she developed paraparesis, and MRI showed a tumefactive longitudinally extensive cervical myelitis. High dose steroids were administered and, subsequently, infliximab was initiated, after which she had clinical and radiological improvement. Patient 9 (19) developed T9–T10 transverse myelitis in the setting of a diffuse systemic inflammatory process, which included uveitis and colitis, after ipilimumab infusions; he had improvement after discontinuation of ICI and high dose steroids. Patient 6 (38) developed a longitudinally extensive transverse myelitis associated with CRMP-5 IgG antibodies and improved with steroids. Even though 5 of 7 patients presented with longitudinally extensive myelitis, none of them fulfilled the criteria for NMOSD. Nevertheless, only two patients were tested for anti-AQP4 (cases 8 and 10); both resulted negative. There was no report of anti-MOG testing for any of the cases.

### Optic Neuritis

We found four case reports (40–43) of isolated optic neuritis associated with ICI. Patients 14 and 17 had been exposed to radiotherapy to treat brain metastases. Patient 15 (43) presented with left eye anterior optic neuropathy associated with aseptic meningitis after ipilimumab treatment and progressed with recurrent bilateral optic neuritis. All four patients had bilateral and anterior optic neuritis, with optic disk swelling. Except for patient 16 (40), who was treated only for associated uveitis with topical steroids, all patients received high dose steroids, and the four had a good outcome. MRI showed optic nerve enhancement in two patients (cases 15 and 17) and was not reported in one (case 16). CSF was only reported in two patients; it was normal in case 16 and revealed pleocytosis in case 15. There was no mention of anti-MOG or anti-AQP4 testing for any of the cases.

### Neuromyelitis Optica Spectrum Disorder

Patient 13 (44) developed a longitudinally extensive tumefactive myelitis after one cycle of nivolumab. CSF showed marked pleocytosis (1,195 cells/mm^3^, 53% neutrophils); although levels this high are uncommon, pleocytosis in NMOSD is usually higher than 50 cells/mm^3^. Based on a positive anti-AQP4, a diagnosis of NMOSD was made. He had no brain or optic nerve involvement and a negative anti-MOG and paraneoplastic panel. Although he had an improvement of MRI lesions after treatment with high dose steroids and PLEX, he had a minimal symptomatic response. Anti-AQP4 testing, performed on the serum collected on the day of nivolumab infusion, was negative. This report suggests seroconversion after ICI treatment and strengthens a causal relationship between nivolumab use and NMOSD.

### Atypical Demyelination

Six patients developed atypical demyelination and did not fit into previous diagnostic groups (14, 25, 46–49). Imaging patterns were typical for MS in two cases, with periventricular white matter lesions with incomplete gadolinium enhancement (patient 19) and small non-enhancing periventricular and spinal cord white matter lesions (patient 21). Even though these two patients had radiologic findings suggestive of a clinically isolated syndrome (CIS) and MS, respectively, we chose to classify them in the atypical demyelination group because of their monophasic presentation and because the authors do not mention those diagnoses being made. The remaining 4 cases had atypical demyelination imaging: cases 18 and 20 presented with tumefactive white matter lesions, case 22 had multiple hyperintense T2 flair signal white matter lesions, and case 23 developed longitudinally extensive myelitis associated with pons and mesial temporal lobe hyperintensities typical for limbic encephalitis. All four patients had focal deficits and altered mental status.

Two cases had reported brain metastasis treated with radiosurgery either after (patient 18) or between (patient 19) ICI infusions. In case 18, one of the several demyelinating lesions bordered the previously irradiated neoplastic lesion. Patients who had a higher volume of T2 hyperintense lesions, including tumefactive (patients 20 and 23) or large lesions (patient 18), had a worse outcome, with no response and death despite steroids and other immunosuppressive treatments, as opposed to patients 19, 21, and 22 who had smaller demyelinating lesions and satisfactory response to steroids and/or discontinuation of ICI.

An autopsy was performed for case 20, which showed white matter widespread demyelination, with infiltration of macrophages containing myelin debris, reactive astrocytes, focal perivascular lymphoid inflammation, and areas of early cavitation. CD8^+^ T cells were seen perivascularly and at the edge of acutely demyelinating plaques, whereas CD4^+^ T cells were confined to perivascular spaces and in smaller numbers (47). In case 18, a biopsy of the lesion that showed acute/subacute demyelination was processed to assess the functional profiles of the patient's T cells. The functional profiles of the patient's myelin-reactive T cells were compared to a T-cell library of MS and healthy controls. Similarly to MS, proliferation rates and pro-inflammatory cytokine production of myelin-reactive CD4^+^ T cells were higher, and anti-inflammatory cytokine IL-10 production was lower than healthy controls, consistent with a T_H_1/T_H_17 immune phenotype (25).

Patient 23 (14) had a strongly positive Anti-Hu antibody in the serum. Although anti-Hu was present before ICI treatment, he developed symptoms only after receiving nivolumab, which suggests the role of checkpoint blocking on the development of the paraneoplastic syndrome. He presented with an anti-Hu associated encephalomyelitis, with temporal lobe involvement suggestive of limbic encephalitis and longitudinally extensive cervical myelitis with imaging consistent with demyelination. The postmortem autopsy findings showed that microglia were highly expressed in the hippocampus, pons, and spinal cord, and CD8-positive T cells and macrophages invaded the medial aspect of the temporal lobe, thalamus, cerebellum, and spinal cord.

## Discussion

Immune checkpoint molecules appear to play a critical role in tolerance to self-antigens and have been implicated in several immune-mediated disorders (51). Some of the proposed mechanisms by which general immunological adverse events occur with the use of ICI include (1) a shift toward the pro-inflammatory profile of T lymphocytes dominated by Th1/Th17 differentiation that increases the production of pro-inflammatory cytokines, (2) autoreactive antibody production, (3) activation of potentially pre-existing self-reactive T cells, and (4) a cross-reactivity between normal tissue antigens and tumor neo-antigens (52–54).

Before ICI advent, there were few reports of focal or multifocal white matter demyelination associated with cancer, and the paraneoplastic nature of these findings was not clear (55). The most convincing cases are those associated with seminoma (56–59). Other reports, many of which are associated with lymphoma, are less convincing because of the possibility that brain lymphoma treated with corticosteroids may have interfered with diagnosis (55). There does not appear to be an increased risk of cancer in patients with multiple sclerosis, possibly except for breast cancer (60). Nevertheless, we believe that the cases compiled here are not paraneoplastic *per se*, but rather a complication of the immune response triggered by the ICI treatment, with or without the participation of tumoral antigens.

CNS demyelination can be induced in animal models through the modification of the checkpoint pathways. For example, blocking CTLA-4 in a relapsing–remitting experimental autoimmune encephalomyelitis mice model has been shown to exacerbate clinical disease and inhibit clinical remission through enhanced T cell reactivity to epitopes associated with induction and relapse (61). ICI also upregulates costimulatory T cell activation pathways such as the CD28-B7, which appears to play an important role in the pathogenesis of demyelination (62). The suppression of mechanisms that inhibit those pathways could potentially increase the incidence of demyelinating conditions. The activation of the checkpoint pathways, conversely, can be used to treat immune-mediated disorders. Abatacept, a CTLA4-Ig fusion protein, is approved to treat rheumatoid arthritis and juvenile idiopathic arthritis and has been evaluated in a phase II clinical trial for MS, although failed to show efficacy (63).

In our review, the four cases that underwent anatomopathological studies (14, 22, 25, 47) all had a CD8^+^ T cell predominant infiltrate on the CNS, consistent with a T_H_1 immune response. In case 18, CD4^+^ T cells profiles suggested a pathogenic T cell response against myelin (25). Moreover, further CSF examination in case 4 concluded that T cell antitumor response and the CNS autoimmune response were aimed at different antigens, suggesting a more direct effect of ICI in the development of demyelination than the activation of a paraneoplastic reaction (22). In contrast, in case 23, demyelination and limbic encephalitis were thought to result from the induction of a paraneoplastic response associated with anti-Hu antibodies (14).

We found a median of 6.5 weeks from ICI's start to the onset of the demyelinating event. This delay is in keeping with the timing of irAE due to ICI in general. Usually, irAE are subacute and temporally associated with ICI introduction, with serious adverse events tending to occur days to weeks after treatment initiation, whereas paraneoplastic disorders tend to have a slower evolution (64). Despite this, neurologic irAE have been reported throughout treatment and even after treatment discontinuation (65). In this review, the case with the later appearance of symptoms after immunotherapy was case 5, 43 weeks after introducing pembrolizumab.

Neurologic immune-related adverse events are described to be most commonly seen after a combined checkpoint blockade, with agents targeting both PD1/PD-L1 and CTLA4 pathways (66). Nevertheless, only three patients in this review were exposed to a combination of ICIs (cases 10, 19, and 20), probably because of the less frequent use of combined block in current clinical practice. We could not ascertain if single or double checkpoint blockade had differences in nirAE outcomes because of the small number of cases in our review. Additionally, nine out of the 23 cases had been exposed to radiotherapy directed to CNS or spinal metastases. It is conceivable that exposure of myelin antigens by radiotherapy could have triggered an immune response in combination with ICI. The risk of demyelination with the association of CNS radiotherapy and ICI is unclear and should be further studied.

We believe it is essential to question patients undergoing evaluation for ICI about immune antecedents, including a previous diagnosis of immune-mediated disorders and current or previous symptoms that may be caused by an undiagnosed inflammatory condition, such as paraneoplastic disorders (67). In patients who underwent brain MRI for another reason, even without symptoms of MS, it is also interesting to evaluate whether lesions suggestive of demyelination already existed before ICI treatment, as these patients may be more at risk of developing more severe nirAEs (21). Nevertheless, MS relapses seem to be a rare complication of ICI treatment (21, 68), and case reports presumably could overestimate its incidence because of publication bias. For example, we found only one report of a patient who had MS and remained stable when treated with ipilimumab while receiving interferon-beta (69).

### Treatment and Prognosis

According to the European Society for Medical Oncology (ESMO) clinical practice guideline for the management of immunotherapy-related toxicity (6), the recommended treatment for nirAE is the suspension of ICI, associated with corticosteroids in low-dose for mild to moderate cases, or high doses for severe cases, either intravenously or orally. The guideline suggests using intravenous plasmapheresis or immunoglobulin in specific cases, such as myasthenia gravis and Guillain Barré syndrome, and they consider extrapolating this treatment to severe cases of isolated optic neuritis, myelitis, and cases that meet criteria for NMOSD. This recommendation is based on the indication of these treatments for demyelinating syndromes not related to ICI.

Almost all patients presented in this review (18/23) were treated with immunosuppressors, and most of them had a partial or total improvement of nirAE. Overall, nirAE is treatable, has a good prognosis, and is known to be relatively rare, although some groups may have a higher risk (7). Given this, the risk-benefit of introducing ICI is usually favorable, as they are indicated mostly for advanced cancers or those with poor independent prognosis (1).

The demyelination should be treated according to the current guidelines for each specific syndrome (i.e., MS). Three of four MS cases, for example, improved with interferon and glatiramer. In one case (23), MS treatment was withheld when starting ICI because of the preoccupation that it would interfere with the cancer treatment, and the patient relapsed but had a good outcome after treatment. On the other side, glatiramer was maintained in another case (21), and the patient developed demyelination that was unresponsive to steroids. It is not clear if it is safe to start ICI on MS patients. If decided for ICI, MS patients should be monitored closely, and MS treatment should be carefully discussed.

In this context, although not used by any of the reported cases, Natalizumab would be an attractive drug to treat ICI-related demyelination. It is a monoclonal antibody approved for the treatment of multiple sclerosis. Its mechanism of action consists of blockage of lymphocyte migration through the blood-brain barrier due to its anti-α4 integrin effects. As this mechanism is specific, it is not expected to interact with the therapeutic effects of ICIs. Natalizumab has been used in a patient with limbic encephalitis induced by ICI immunotherapy against small cell lung cancer (70), and possibly could have its indication expanded to cases of atypical demyelination related to ICI, using this same rationale.

Despite the potential biological role of TNF-α blockers in triggering or aggravating demyelination (71), infliximab may be another treatment option in refractory ICI-related demyelination. This drug is already used more widely in ICI-related refractory colitis with good outcomes (6, 72), suggesting that decreasing the pro-inflammatory state associated with TNF-α is useful in treating irAE. Based on that, there is a possibility to generalize these findings to treat other refractory immune adverse events related to ICI. Patients 5 (41) and 17 (33) used infliximab after failure of other medications, with an improvement of neurological symptoms, corroborating this hypothesis.

### Limitations

We chose to perform a review only with case reports of demyelination, a rare complication of ICI treatment, to describe the clinical presentation and outcomes in these patients. Nevertheless, there is an inherent limitation of extracting data from reports, which can sometimes be incomplete or lacking a description of investigations that could change the data's interpretation. We feel, though, that most of the cases were well reported. A prospective study, which would be ideal, is difficult in rare complications such as these. Pharmacovigilance reporting as in Food and Drug Administration Adverse Event Reporting System (FAERS) database or European databases is an interesting method to collect prospective data on adverse events of medications. However, currently, the data found there are, in general, not as detailed as case reports.

Since we did not have contact with the patients reported, it is difficult to extrapolate a diagnosis beyond what is mentioned by the authors in the original papers. That is why we included patients 19 and 21 in the atypical demyelination group, despite somewhat typical imaging for CIS or MS. In the same line, one could argue that some of the atypical demyelination cases could be classified as ADEM. Although acute demyelinating encephalomyelitis (ADEM) is well defined in children (31), in adults it does not appear to be a homogeneous entity and remains to be better understood (32). Diagnostic criteria for ADEM in adults have been proposed (32), but they have not yet been validated in larger studies. In our review, cases 18, 20, and 22 had ADEM features such as atypical demyelinating lesions, confusion, and generalized slowing on EEG (patient 22), although two of them (cases 20 and 22) had positive OCB, which is usually absent in ADEM. Since the authors did not mention this diagnosis, we chose to classify them merely as atypical demyelination.

## Conclusions

Demyelination is a rare complication of ICI treatment. Although potentially severe, it is treatable, and outcomes after immunosuppression seem favorable in most patients. At first glance, cases with a higher demyelinating disease burden (i.e., higher lesions volume) appear to have had a worse prognosis. Considering the four cases that underwent pathological examination, we hypothesize that a T_H_1 immune response is possibly the mechanism by which these patients develop demyelination (14, 22, 25, 47). Furthermore, we speculate that the ICI treatment, in addition to improving the T cell immune response against the tumor, may trigger the effector functions of T-cell clonotypes directed toward myelin epitopes (22). Further studies are needed to determine the exact pathophysiology of demyelination associated with ICI and the best treatment for these cases. Natalizumab appears to be a promising treatment candidate and remains to be tested.

## Author Contributions

MO and MS contributed in the conception and design of the study. MO and MB organized the database, performed the statistical analysis, and wrote the first draft of the manuscript. MO, MB, and MS wrote sections of the manuscript. All authors contributed to manuscript revision, read, and approved the submitted version.

## Conflict of Interest

The authors declare that the research was conducted in the absence of any commercial or financial relationships that could be construed as a potential conflict of interest.
