# Supplementary material for: Central Nervous System Demyelination Associated With Immune Checkpoint Inhibitors: Review of the Literature
Source: Front Neurol. 2020 Dec 11;11:538695. doi: 10.3389/fneur.2020.538695 (PMC7759512; doi:10.3389/fneur.2020.538695)
Supplement: Supplementary file 2 [file Data_Sheet_2.PDF]

Supplementary Material

1 Supplementary Figures

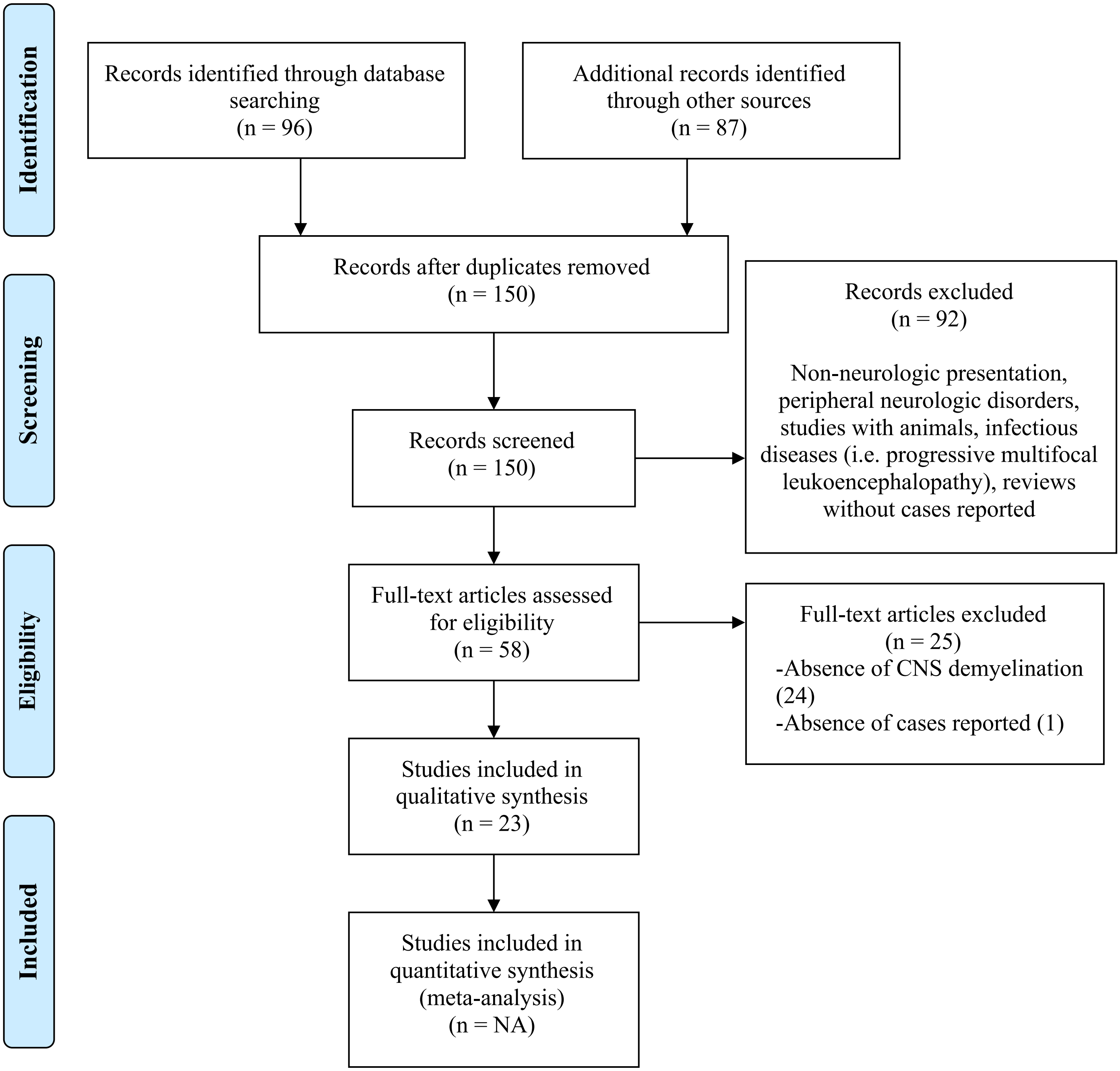

Supplementary Figure 1. Study methodology flowchart (PRISMA flow diagram).
